# Clinical and prognostic aspects of patients with the Neuromyelitis Optica Spectrum Disorder (NMOSD) from a cohort in Northeast Brazil

**DOI:** 10.1186/s12883-022-02621-5

**Published:** 2022-03-16

**Authors:** Thiago Gonçalves Fukuda, Ivã Taiuan Fialho Silva, Tayla Samanta Silva dos Santos, Marcos Baruch Portela Filho, Fernanda Ferreira de Abreu, Jamary Oliveira-Filho

**Affiliations:** 1 Post-Graduate Program in Health Sciences, Federal University of Bahia (UFBA), Salvador, Bahia, Brazil; 2 Neurology Service, Professor Edgar Santos University Hospital, Federal University of Bahia (UFBA), Salvador, Bahia, Brazil; 3 State University of Bahia (UNEB), Salvador, Bahia, Brazil

**Keywords:** Neuromyelitis optica spectrum disorders, Prognostic factors, Brazilian Northeast, Aquaporin-4

## Abstract

**Introduction:**

Neuromyelitis optica spectrum disorders (NMOSD) is a rare inflammatory and demyelinating disease of the central nervous system (CNS) more frequent in women and Afro-descendants. No previous epidemiological or prognostic study has been conducted in the region of the state of Bahia, Brazilian Northeast.

**Objective:**

To evaluate clinical and prognostic aspects in patients with NMOSD from a cohort in northeastern Brazil.

**Material and methods:**

A single-center retrospective study was conducted with consecutive patients diagnosed with NMOSD. Clinical and epidemiological characteristics were described. The degree of disability was expressed by the Expanded Disability Status Scale (EDSS). Worsening disability were analyzed through negative binomial regression adjusted for disease duration.

**Results:**

Ninety-one patients were included, 72 (79.1%) female and 67 (73.6%) afro descendants. Mean age at onset was 36 (± 14) years and 73.3% were anti-aquaporin-4 antibody positive. Isolated transverse myelitis (32.9%) and isolated optic neuritis (22.4%) were the most frequent initial clinical syndromes. After multivariate analysis, optic neuritis (*RR* = 0.45; 95% CI = 0.23 – 0.88; *p* = 0.020) and dyslipidemia (*RR* = 0.40; 95% CI = 0.20 – 0.83; *p* = 0.014) were associated with slower disease progression. Area postrema involvement (*RR* = 6.70; 95% CI = 3.31 – 13.54; *p* < 0.001) and age at onset (RR = 1.03; 95% CI = 1.01 – 1.05; *p* = 0.003) were associated with faster disease progression.

**Conclusions:**

In the first clinical and prognostic study in northeastern Brazil, we identified area postrema involvement, age at onset, optic neuritis at fist syndrome and dyslipidemia as the main prognostic factors associated with disease progression.

**Supplementary Information:**

The online version contains supplementary material available at 10.1186/s12883-022-02621-5.

## Highlights

• Isolated transverse myelitis was the most frequent initial clinical syndrome.

• Optic neuritis as first clinical syndrome was independently associated with better prognostic.

• Age at onset were associated with faster disease progression.

• Hashimoto's thyroiditis was the most frequent autoimmune disease in NMOSD.

**Supplementary Information:**

The online version contains supplementary material available at 10.1186/s12883-022-02621-5.

## Introduction

Neuromyelitis optica spectrum disorders (NMOSD) is an inflammatory and demyelinating disease of the central nervous system (CNS). The pathophysiology of NMOSD results from the autoimmunity against water channels (aquaporin-4) that are widely distributed in the CNS [1, 2]. The most recent diagnostic criteria recognize six core clinical characteristics of presentation during an attack, which the most common are optic neuritis (ON), longitudinally extensive transverse myelitis (LETM) and area postrema syndrome (APS) [3].

The natural history of the disease is variable, with most cases presenting a relapsing remitting course. It leads to accumulation of neurologic deficits from attacks with slow or partial recovery, which results in functional disability due to visual and motor sequelae [4]. Because of the variability and severity within the course of the disease, the ability to predict risk factors associated with poor outcomes can be useful to help physicians choosing therapeutic approaches.

NMOSD is a rare disease with an estimated prevalence of 0.3–4.4/100,000, more frequent in women and Afro-descendants [5]. Few epidemiological studies have been carried out in Europe, Asia, the United States and Latin America [6–12]. No studies were found in northeastern Brazil or in the State of Bahia, State with the highest percentage of blacks outside Africa [13].

Previous studies have evaluated epidemiologic and clinical features as predictors of disease outcomes. Optic neuritis as initial clinical presentation has been associated with poor visual acuity outcomes, whereas the seropositive to AQP4-IgG and poor recovery from the first attack have been shown to predict greater severity in the course of the disease [8, 14, 15]. Other known predictors of worse prognosis include age at disease onset, afro descendent ethnicity, higher number of attacks before and after immunosuppressive treatment and association with other autoimmune diseases [14–19].

In the present study, we aim to evaluate clinical, demographic, radiologic and laboratory data from 91 patients in order to foresee possible features of worst disease progression.

## Material and methods

### Study design

A retrospective study was conducted in which 91 patients with NMOSD were included, based on 2015 criteria [3], attended consecutively between 2017 and 2019. All patients were followed-up at the NMOSD and MS references center from Federal University of Bahia, located in northeastern Brazil. This is the only reference center in the state of Bahia.

All patients who met the criteria and agreed to participate in the study signed the consent form that includes access to medical records by researchers. For patients under the age of 18 was also signed by a guardian.

### Data

Data were obtained prospectively through an interview on the day of the outpatient consultation and complemented by retrospective review of medical records through a standardized questionnaire applied by the group of researchers. Clinical, demographic, laboratory and MRI data were collected from all patients who met the inclusion criteria.

The following factors were evaluated: age at onset, sex, ethnicity, educational level, first syndrome at onset, number of attacks, annualized relapse rate, disease sequelae, co-morbidities, concurrent autoimmune diseases, and AQP4-IgG status. Disease duration was defined as the time interval between disease presentation and last follow-up. Delay until diagnosis was considered when the time between the onset of symptoms and diagnosis was greater than 12 months.

The information related to MRI analysis was obtained through a report provided by neuroradiologist based on the first examination performed after the onset of symptoms. Information was collected based on topography of brain, and spinal cord lesion (cervical, thoracic, and lumbar) and extension of spinal cord lesion (number of vertebral segments). Lesions in the area postrema were analyzed separately from the other medulla oblongata topographies. We defined longitudinally extensive myelitis when the lesion affected more than three continuous vertebral segments.

The degree of disability was quantified by the Expanded Disability Status Scale (EDSS) [20] applied at the time of patient admission to the study by a certified neurologist, EDSS scoring was performed at least 30 days after last attack.

To control bias due to the inclusion of patients with different disease durations, parameters such as progression index, annualized relapse rate and negative binomial regression were used.

Progression index [21] was defined by the ratio between EDSS and the duration of the disease, whereas annualized relapse rate was defined as the ratio between the number of attacks and the disease duration in years.

### Statistical analysis

Statistical analysis was performed using STATA Statistical Software version 14.1 (StataCorp, College Station, TX). Categorical data was described in simple frequencies and proportions (%). Continuous normally distributed variables were described as means and standard deviations; and median and interquartile range (IQR) for those with non-normal distribution. The distribution of quantitative variables was observed by visual inspection and with the Kolmogorov–Smirnov test.

We used negative binomial regression to assess the association between each independent variable to the outcome of disability progression over time (EDSS as the dependent variable and disease duration in months as the offset variable). Variables with a possible association in univariable analyses (*p* < 0.1) were included in a final multivariable negative binomial regression model, with was used to estimate relative risks (RR) and 95% confidence intervals. Statistical significance was set at an alpha level of 5%.

## Results

### General description of the sample (Table 1)

**Table 1:** General characteristics of 91 patients with neuromyelitis optica spectrum disorders

Characteristics	Total
**Age at onset (years)**, average ± SD	36.3 ± 13.6
**Female**, n (%)	72 (79.1)
**Afro-descendants**, n (%)	67 (73.6)
**First syndrome**, n (%)	
Optic neuritis	19 (22.4)
Transverse Myelitis	28 (32.9)
Area postrema syndrome	5 (5.9)
ON + TM	18 (21.2)
ON + TM + APS	5 (5.9)
ON + APS	3 (3.5)
TM + APS	7 (8.2)
**EDSS**, median, (interquartile)	4 (2.62–6.50)
**AQP4-IgG + **, n (%)	55 (73.3)*
**Autoimmune disease**, n (%)	9 (10.2)
**Recurrence**, n (%)	71 (83.5)
**Number of attacks**, average ± SD	4.0 ± 3.6
**Disease time (years)**, average ± SD	7.8 ± 6.9
**Relapse rate**, average ± SD	1.3 ± 1.6
**Progression index**, average ± SD	2.3 ± 4.1
**Brain MRI lesions**, n (%)	57 (75.0)
**Spinal cord MRI lesions**, n (%)	68 (90.7)
Number of affected vertebral bodies, median, (interquartile)	5 (3.0–7.0)
**Longitudinally extensive lesion**, n (%)	51 (78.5)
**Spinal cord lesion topography**, n (%)	
Cervical	25 (38.5)
Thoracic	10 (15.4)
Cervical and thoracic	18 (27.7)
Thoracic and lumbar	2 (2.9)
Lumbar and sacral	1 (1.5)
Without injury	9 (13.8)
**Type of treatment,** n (%)	
Azathioprine and glucocorticoid	37 (50.7)
Azathioprine	30 (41.1)
Rituximab	6 (8.2)

*ON* Optic Neuritis, *TM* Transverse Myelitis, *APS* Area postrema syndrome

^*^We had 55/75 (73.3% of those tested) AQP4-positive patients, 20/75 (26.6% of those tested) patients with NMOSD Aquaporin 4 negative and 16 met diagnostic criteria but had unknown aquaporin status (not tested or results not yet available)

Ninety-one outpatients were included in this study, 72 (79.1%) were female and 67 (73.6%) afro descendants. The mean age at the interview was 45 (± 14) years, while the mean age at onset of the disease was 36 (± 14) years. The other sociodemographic data can be found in Table 1.

Among the clinical syndromes presented in the first event, isolated transverse myelitis (32.9%) and isolated optic neuritis (22.4%) were the most frequent, followed by ON and TM simultaneously (21.2%) and area postrema syndrome (5.9%). In the 75 outpatients with information on anti-aquaporin-4 antibody (AQP4) status, 73.3% were positive. Of the aquaporin-4 negative patients we tested only 7 patients; two additional patients with seropositivity only for Anti-MOG were excluded from the analysis. No patients positive for aquaporin 4 were tested. The median EDSS at the first interview was EDSS of 4.0 [interquartile range (IQR): 2.62–6.50]. The recurrent course was more frequent (83.5%), and the average number of attacks was 4.0 (± 3.6). The mean annualized relapse rate was 1.3 (± 1.6) and the mean progression index was 2.3 (± 4.1).

The most frequent comorbidity was systemic arterial hypertension (29.8%), followed by depression (24.4%), dyslipidemia (22.0%), diabetes mellitus (15.7%) and smoking (10.8%). Other autoimmune diseases were present in nine (10.2%) patients, such as Hashimoto's thyroiditis (four patients), systemic lupus erythematosus (three patients), Sjögren’s syndrome (one patient) and vitiligo (one patient).

Brain lesions were found in 52/73 (71.2%) patients who underwent brain magnetic resonance imaging (MRI). Most lesions were periventricular (17.8%), followed by area postrema (16.4%) and in the cerebellum (13.7%). In 65 patients who underwent spinal MRI, 51 (78.5%) had a longitudinally extensive lesion. The topography most affected was cervical (38.5%), followed by cervical and thoracic involvement (27.7%) and only thoracic (15.4%). The median number of affected vertebral bodies was 5.0 (IQR: 3.0–7.0).

Comparison of demographic, clinical and radiological characteristics between NMOSD patients with positive and negative aquaporin 4 status was performed and no statistically significant difference was found (Supplementary Table 1).

### Association of age of onset of symptoms and worse rate of progression (Fig. 1)

**Fig. 1:**
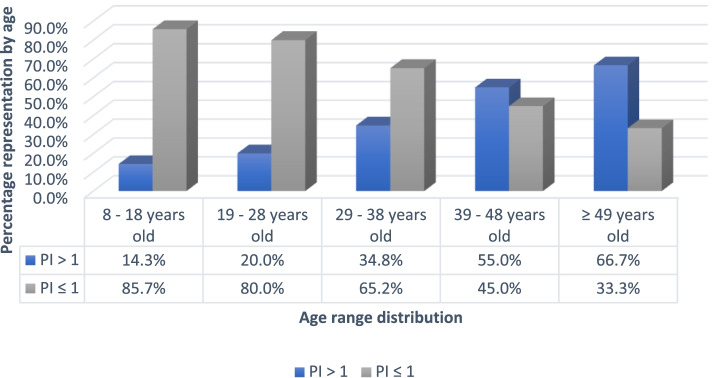
Percentage distribution of the progression index by age group at onset of symptoms

When comparing patients in terms of progression index, older age at disease onset was seen in fast (> 1 per year) vs slow (≤ 1 per year) progression index (43.0 ± 13.8 vs 33.3 ± 11.5 years of age, respectively). Similarly, three other findings were more frequent among patients with fast vs slow progression index: transverse myelitis as the initial presentation of the disease (50.0% vs 21.7%), diagnosis of autoimmune disease (22.6% vs 4.3%) and area postrema lesions (33.3% vs 2.7%).

### Univariable predictors of worse disability progression over time (Table 2)

**Table 2:** Univariable predictive clinic factors for early progression in neuromyelitis optica (negative binomial regression)

Variable	Relative risk	95% confidence interval	*P*-value
** Sex**			
Male	1.61	0.30 – 8.63	0.578
Female	Reference	–	–
** Skin color**			
Black	0.757	0.16 – 3.58	0.726
Others	Reference	–	–
** Age at onset (years)**	1.07	1.01 – 1.12	**0.020**
** Co-morbidities**			
Autoimmune disease	3.37	0.45 – 25.50	0.239
Hypertension	0.90	0.20 – 4.16	0.896
Diabetes mellitus	0.40	0.05 – 3.16	0.383
Dyslipidemia	0.09	0.18 – 0.51	**0.006**
Depression	0.20	0.04 – 1.12	0.067
Smoking	0.56	0.06 – 4.90	0.597
** First syndrome**			
Optic neuritis	0.06	0.01 – 0.44	**0.006**
Area postrema	0.38	0.02 – 6.28	0.497
Optic neuritis + transverse myelitis	0.10	0.01 – 0.66	**0.017**
Others	0.94	0.30 – 2.92	0.917
Transverse myelitis	Reference	–	–
** AQP4-IgG + **	1.26	0.24–6.59	0.781
** Number of brain injuries on MRI**	1.13	0.66–1.92	0.657
** Topography of brain lesions on MRI**			
Midbrain	1.07	0.00–5.97	0.276
Pons	2.10	0.24–18.08	0.500
Medulla Oblongata	2.45	0.37–16.04	0.349
High cervical spinal cord	2.94	0.39–22.35	0.298
Cerebellum	0.82	0.10–6.69	0.852
Periventricular	4.35	0.69–27.66	0.119
Area Postrema	6.55	0.98–43.58	**0.052**
** Optic nerve MRI lesions**	0.13	0.00–8.71	0.346
** Delay until diagnosis (> 12 m)**	12.53	3.12–50.26	< 0.001

Older age at disease onset was associated with worse disability progression [*RR* = 1.07; 95% CI (1.01–1.12); *p* = 0.020]. Patients with dyslipidemia had a milder progression [*RR* = 0.09; 95% CI (0.18–0.51); *p* = 0.006].

Regarding the core syndrome of the first attack, outpatients who were affected only optic neuritis had a slower disability progression when compared to those who had only transverse myelitis [*RR* = 0.06; 95% CI (0.01–0.44); *p* = 0.006]. Similarly, those who had transverse myelitis and optic neuritis simultaneously at the first attack had slower progression [*RR* = 0.10; 95% CI (0.01–0.66); *p* = 0.017] when compared to those who had only transverse myelitis. Delay until diagnosis was associated with worse prognosis [*RR* = 12.53; 95% CI (3.12–50.26); *p* < 0.001].

There was no difference in progression regarding the positivity of the AQP4-IgG antibody [*RR* = 1.26; 95% CI (0.24–6.59); *p* = 0.781]. Outpatients with lesions in the area postrema tended to worse disability progression [*RR* = 6.55; 95% CI (0.98–43.58); *p* = 0.052].

### Multivariable analysis (Table 3)

**Table 3:** Multivariable predictors of early progression in optic neuromyelitis (negative binomial regression)

Variable	Relative risk	95% confidence interval	*P*-value
Optic neuritis at first syndrome	0.45	0.23 – 0.88	0.020
Area postrema involvement	6.70	3.31 – 13.54	< 0.001
Dyslipidemia	0.40	0.20 – 0.83	0.014
Depression	1.14	0.56 – 2.32	0.702
Age at onset (years)	1.03	1.01 – 1.05	0.003
Delay until diagnosis (> 12 m)	1.14	0.56 – 2.30	0.712

In the multivariable analysis, optic neuritis [*RR* = 0.45; 95% CI (0.23 – 0.88); *p* = 0.020] and dyslipidemia [*RR* = 0.40; 95% CI (0.20 – 0.83) *p* = 0.014] were associated with slower disability progression. Area postrema lesion [*RR* = 6.70; 95% CI (3.31 – 13.54); *p* < 0.001] and age at onset [*RR* = 1.03; 95% CI (1.01 – 1.05); *p* = 0.003] were associated with worse disease progression.

## Discussion

This study is the first to evaluate clinical and demographic features in the Brazilian northeast region. Although our study is not population-based, we believe that we were able to investigate a significant proportion of patients with NMOSD in the state of Bahia since our study center is the only reference center in the state.

Despite methodological differences and disparities in the definition of NMOSD over the years, we have a similar average age at onset compared to most studies, as well as the higher prevalence in women, with female: male proportions > 3:1[6–8, 10, 22–24]. Our sample has a higher percentage of afro-descendant patients (73.6%) compared to other international studies and even compared with other studies in Brazil. [6–8, 10, 22–24]. This could be due to the population profile of the study location, the city of Salvador, state of Bahia, which concentrates the highest proportion of black people outside Africa [13]. The higher prevalence of NMOSD among Afro-descendant populations explains the sample size of this study, which is comparable to prior multicenter studies that evaluated epidemiological aspects of entire countries [10, 11].

Despite this peculiarity, we did not find a significant association between the race of patients and worst clinical outcomes. This association shows a great divergence in the literature. A previous study demonstrated that race is not an independent factor of worse motor or visual prognosis [17]. Another author demonstrates that there is an association between the white race and worse visual outcomes and higher EDSS scores [14]. In addition, some papers found worse prognosis in Afro-Caribbean patients [18, 25]. Due to the wide miscegenation of the population it’s difficult to define race in this Brazil, thus the racial stratification analyzed in this study was based on the patients’ phenotype.

The guideline of the Brazilian Academy of Neurology, which is the main representative entity of neurologists in Brazil, on NMOSD is currently in progress. There is a manual published by this entity in 2016 that guides as first line—first option (rituximab, azathioprine or azathioprine plus corticoids), first line—second option (mycophenolate) and second line (methotrexate and cyclosporine) [26]. All our patients were using first line, first option medications.

The AQP4-IgG seropositivity was observed in 73.3% of patients, in accordance with prior studies [6, 7, 22, 24]. Other authors found a lower frequency of AQP4-IgG seropositivity [8, 11]. This divergence can be attributed to the methodology used and the timing of the antibody test. Although in some studies the seropositivity of AQP4-IgG antibody has been associated with more severe impairment [15, 27, 28]. Others, similar to our results, showed no association [11, 22].

According to many studies transverse myelitis was the most prevalent syndrome of initial attack (32.9%) [7, 10, 29]. The classic presentation of simultaneous ON and TM was observed in 21.2% of the patients, a percentage that is in agreement with other samples [7, 8, 22]. However, it was smaller than the percentage observed in other cohorts [6, 10]. Another important feature of our sample is that 5.9% of the patients had ON, TM and APS simultaneously in the initial presentation. This data can be explained by another study that demonstrated that combined initial clinical syndromes are more frequent in Afro-American and Afro-European populations [17].

With regards to comorbid autoimmune diseases, the frequency in our sample is similar (10.2%) to what was shown in some previous studies [6, 8]. When compared to others studies, however, where up to a third of patients presented this type of comorbidity, our percentage was lower [10, 11, 22]. In our study, we observed that Hashimoto's thyroiditis was the most frequent autoimmune disease, followed by systemic lupus erythematosus, comorbidities that were previously associated with NMOSD [30]. Furthermore, the percentage of comorbid autoimmune diseases tended to be higher in patients with disease progression, but this association was not statistically significant in the multivariate analysis. A prior study described an association between concomitance of other autoimmune diseases and greater disability [31].

The annualized relapse rate as well as the frequency of relapsing remitting phenotype among patients were similar to the described in the literature. However, we found a progression index of 2.3 (± 4.1), an index higher than the one reported in a previous southeastern Brazilian study [8]. Regarding the treatment options, the most frequent type of maintenance therapy used was the combination of azathioprine and glucocorticoid, which is accordant to other studies [8, 11, 22].

Concerning the prognostic factors, a higher age at the initial symptomatic presentation was associated with worst clinical outcome, as reported previously [32, 33]. Our findings also suggest that patients who had area postrema lesions could have worst disease outcomes. Previous studies indicate the medulla oblongata is a highly frequent CNS structure involved in NMOSD (prevalence varying between 12.8% and 91.3%), with lesions usually occurring in its dorsal part [34–37]. One study demonstrated that patients with medullary lesions were more likely to have a higher number of brain injuries, as well as disease with elevated clinical activity and rapid progression, hence reporting higher EDSS and annualized relapse rate scores [38]. To explain this more severe behavior among patients with area postrema injuries, we speculate that: 1) the signs and symptoms resulting from lesions in this topography can more severely increase the EDSS; 2) these patients are more often affected by a greater number of brain and extensive spinal cord injuries, which could have an impact on the severity of the disease.

Although it is not clear if the initial clinical syndrome type has an influence on prognosis, our study found that optic neuritis as first presentation was associated with slow disease progression. A recent retrospective study demonstrated that optic neuritis as initial presentation of NMOSD correlated to worst visual outcome, with no differences regarding EDSS [14]. One study showed that patients with optic neuritis as an initial symptom of multiple sclerosis took more years to achieve EDSS 4.0, 6.0 and 7.0 when compared to patients without neuritis [39]. Similarly, another study reported that patients who had optic neuritis as initial attack (clinically isolated syndrome) had a lower rate of conversion to multiple sclerosis, when compared to other types of first disease presentation [40]. However, this type of optic neuritis differs from atypical optic neuritis, as generally seen in NMOSD and further prospective studies are needed to investigate the real influence of the initial syndrome on the prognosis of patients. One of the limitations presented by the study was the low percentage of patients tested for anti-MOG and a possible association of patients with ADEM-like phenotype and/or concomitant neuritis and myelitis not being associated with a worse prognosis.

One of the most unexpected aspects of the present study was the finding of dyslipidemia as a factor associated to a better prognosis, a feature that was not analyzed in most of the previous epidemiological cohorts. This result is contrary to a previous observation that hypertriglyceridemia may be related to a worse recovery from the first demyelinating NMOSD attack [41]. One of the theories suggested to explain this finding is that dyslipidemic patients may have used hypolipidemic drugs as a long term treatment, such as statins, that have pleotropic anti-inflammatory effects and, therefore, a possible beneficial effect on disease control [42]. A second possible explanation is that the inflammatory activity is associated with reduction in LDL levels, as observed in other inflammatory diseases, such as rheumatoid arthritis, and in acute stress conditions, such as sepsis [43]. Thus, this could disguise the diagnosis of dyslipidemia in patients with greater inflammatory activity.

## Conclusions

This is the first article to evaluate clinical, epidemiological and prognostic factors in the Brazilian northeast region, in which we assume concentrate an elevated prevalence of NMOSD, due to its ethnical singularities. Area postrema involvement and age at onset were independently associated with greater disability and optic neuritis at fist syndrome and dyslipidemia with less disease progression. Nevertheless, the prognostic aspects here reported need to be confirmed by prospective studies.

## Supplementary Information

**Additional file 1:** **Supplementary Table 1.** Comparison of demographic, clinical and radiological characteristics between patients with NMOSD with positive and negative aquaporin status 4.

## Data Availability

The datasets used and/or analyzed during the current study available from the corresponding author on reasonable request. Please contact TGF at thiagofukuda@gmail.com if this information is of interest.
