# Supplementary material for: Clinical and prognostic aspects of patients with the Neuromyelitis Optica Spectrum Disorder (NMOSD) from a cohort in Northeast Brazil
Source: BMC Neurol. 2022 Mar 16;22:95. doi: 10.1186/s12883-022-02621-5 (PMC8925163; doi:10.1186/s12883-022-02621-5)
Supplement: Supplementary file 1 — Additional file 1: Supplementary Table 1. Comparison of demographic, clinical and radiological characteristics between patients with NMOSD with positive and negative aquaporin status 4. [file 12883_2022_2621_MOESM1_ESM.docx]

| **Characteristics** | **Aqp4- (n=20)** | **Aqp4+ (n=55)** | ***p-value*** |
| --- | --- | --- | --- |
| **Age at onset (years)**, *average ± SD* | 38.3±13.4 | 36.0±14.0 | 0.518 |
| **Female**, *n (%)* | 16 (80.0) | 46 (83.6) | 0.737 |
| **Afro-descendants**, *n (%)* | 16 (80.0) | 39 (70.9) | 0.560 |
| **First syndrome**, *n (%)* |  |  |  |
| Optic neuritis | 6 (30.0) | 10 (18.9) | 0.058 |
| Transverse Myelitis | 4 (20.0) | 17 (32.1) |  |
| Area postrema syndrome | 4 (20.0) | 1 (1.90) |  |
| ON + TM | 4 (20.0) | 14 (26.4) |  |
| ON + TM + APS | 0 (0.0) | 5 (9.4) |  |
| ON + APS | 0 (0.0) | 3 (5.7) |  |
| TM + APS | 2 (10.0) | 3 (5.7) |  |
| **EDSS**, *median, (interquartile)* | 3 (1.5-4.5) | 4 (2.7-6.7) | 0.150 |
| **Autoimmune disease***, n (%)* | 2 (10.0) | 5 (9.1) | 1.000 |
| **Recurrence***, n (%)* | 16 (84.2) | 46 (83.6) | 1.000 |
| **Number of attacks**, *average ± SD* | 3.4±3.1 | 4.6 ±3.9 | 0.230 |
| **Disease time (years)**, *average ± SD* | 6.4 ±5.6 | 8.4 ±7.0 | 0.251 |
| **Relapse rate**, *average ± SD* | 1.5 ±1.7 | 1.3 ±1.5 | 0.697 |
| **Progression index**, *average ± SD* | 2.2 ±4.7 | 2.2 ±3.9 | 0.975 |
| **Brain MRI lesions**, *n (%)* | 11 (68.8) | 35 (74.5) | 0.747 |
| **Spinal cord MRI lesions**, *n (%)* | 17 (94.4) | 41 (87.2) | 0.663 |
| Number of affected vertebral bodies, *median, (interquartile)* | 3 (2-5) | 5 (3-7) | 0.109 |
| **Longitudinally extensive lesion**, *n (%)* | 11 (68.8) | 31 (79.5) | 0.279 |
| **Spinal cord lesion topography**, *n (%)* |  |  |  |
| Cervical | 9 (60.0) | 14 (35.0) | 0.666 |
| Thoracic | 2 (13.3) | 8 (20.0) |  |
| Cervical and thoracic | 2 (13.3) | 9 (22.5) |  |
| Thoracic and lumbar | 0 (0.0) | 1 (2.5) |  |
| Lumbar and sacral | 0 (0.0) | 1 (2.5) |  |
| Without lesion | 2 (13.3) | 7 (17.5) |  |
| **Type of treatment,** *n (%)* |  |  |  |
| Azathioprine and glucocorticoid | 10 (52.6) | 28 (56.0) | 0.729 |
| Azathioprine | 8 (42.1) | 17 (34.0) |  |
| Rituximab | 1 (5.3) | 5 (10.0) |  |

**Supplementary Table1.** Comparison of demographic, clinical and radiological characteristics between patients with NMOSD with positive and negative aquaporin status 4.

ON = Optic Neuritis; TM = Transverse Myelitis; APS = Area postrema syndrome
